# Remarks on Puerperal Convulsions

**Published:** 1813-08

**Authors:** 


*To the Editors of the Medical and Physical Journal.*

GENTLEMEN,

I SHALL take the liberty of making a few remarks on a case, at page 36 of your last Number, dignified with the name of *Convulsions during Labour,* because no proof is adduced that this was a case of puerperal convulsions, and because I think that the *prompt* and *decisive* mode of treatment, which your correspondent takes credit to himself for adopting, was very unsuitable to that complaint.

Let us inquire what are the diagnostic symptoms of convulsions during labour?

1st. These are commonly some of the *precursory* symptoms, viz. giddiness or severe pain in the head; bloated or suffused countenance; vacillation of mind; temporary loss of sight or indistinctness of vision; drowsiness, or heaviness to sleep; sometimes a very slow pulse.

2dly. The *immediate* symptoms are those of the epileptic paroxysm. The patient suddenly loses all sensation; the muscles first become extremely rigid, and are speedily afterwards thrown into violent convulsions; the face is distorted; the the eyes are protruded; the patient gnashes her teeth, and foams at the mouth. After this paroxysm is over, she remains in a comatose state, has stertorous breathing, and at length, except in very aggravated cases, slowly recovers her recollection. After a longer or shorter interval, a fresh attack takes place, and it is to be remarked, that a case of true puerperal convulsions very rarely happens in which there is not a repetition of the paroxysm.

None of these symptoms are mentioned by your correspondent as being present in his patient's case: we are only told that during his absence she had been attacked with convulsions, and that about an hour afterwards he found "her extremities contracted and stiff, her pulse nearly gone, her breathing scarcely perceptible, and a peculiar wildness in her countenance," symptoms by no means indicative of the access of puerperal convulsions, an hour before; on the contrary, at this period after the convulsions, the pulse will be found beating with great force, and the breathing will be hard and laborious.

It may be asked, if the patient mentioned at p. 36 had not puerperal convulsions, what was her case? A slight attention to the narrative will, I conceive, resolve this question. We are told that the poor woman was in a state of despondency on account of the circumstances of a former labour, in which it had been necessary to have recourse to the forceps. This state of mind just fitted her for an attack of *hysteria*, and this, and not puerperal convulsions, was, I conceive, her disease. Should this, however, be doubted, it will be sufficient to refer the incredulous to the *prompt* and *decisive* mode of treatment which effected the cure, viz. *two tea cupfuls of a strong mixture of brandy and water.* We have the authority of every old woman in the kingdom that brandy is a sovereign remedy for hysteric symptoms and faintings, but there are few practitioners who would chuse to rely upon this powerful stimulant as a means of curing puerperal convulsions. I remain, Gentlemen,

Your occasional Correspondent,

*July* 10, 1813. OBSTETRICUS.

P. S. Your correspondent, Dr. Spark, p. 29, would confer a great obligation on practitioners of midwifery, particularly the younger ones, if he would point out a mode of readily distinguishing between the cases of tedious labour arising from spasmodic affection of the uterus, and those arising from rigid muscular fibre; they will, I fear, very often mistake the one for the other. Dr. S. imagines that the opium in the first case will have the happiest effects, in the the latter none. Now I cannot conceive of opium that if it is not a very efficacious drug, it will prove altogether inert: I think if it does not do good, it must do mischief, in the large doses which Dr. S. recommends: hence the necessity of pointing out with great precision the cases in which it may be safely employed.

